# Combinatorial ECM Arrays Identify Cooperative Roles for Matricellular Proteins in Enhancing the Generation of TH+ Neurons From Human Pluripotent Cells

**DOI:** 10.3389/fcell.2021.755406

**Published:** 2021-12-01

**Authors:** Maqsood Ahmed, Matthew J. S. Owens, Enrique M. Toledo, Ernest Arenas, Mark Bradley, Charles ffrench-Constant

**Affiliations:** ^1^ Centre for Regenerative Medicine, University of Edinburgh, Edinburgh, United Kingdom; ^2^ School of Chemistry, EaStCHEM, University of Edinburgh, Edinburgh, United Kingdom; ^3^ Laboratory of Molecular Neurobiology, Department of Medical Biochemistry and Biophysics, Karolinska Institutet, Stockholm, Sweden; ^4^ Faculty of Medicine and Health Sciences, University of East Anglia, Norwich, England

**Keywords:** extracellular matrix, neural stem cells, tyrosine hydroxylase, matricellular proteins, dopaminergic (DA) neuron, array

## Abstract

The development of efficient cell culture strategies for the generation of dopaminergic neurons is an important goal for transplantation-based approaches to treat Parkinson’s disease. To identify extracellular matrix molecules that enhance differentiation and might be used in these cell cultures we have used micro-contact printed arrays on glass slides presenting 190 combinations of 19 extracellular matrix molecules selected on the basis of their expression during embryonic development of the ventral midbrain. Using long-term neuroepithelial stem cells (Lt-NES), this approach identified a number of matricellular proteins that enhanced differentiation, with the combination of Sparc, Sparc-like (Sparc-l1) and Nell2 increasing the number of tyrosine hydroxylase+ neurons derived from Lt-NES cells and, critically for further translation, human pluripotent stem cells.

## Introduction

The generation of large numbers of dopaminergic neurons from human pluripotent stem cells is an important goal for therapeutic strategies based on transplantation to replace these cells in Parkinson’s disease (Parmar et al., 2020). Such strategies have shown promise in some patients transplanted with human fetal cells (Barker et al., 2013), and larger scale studies are currently underway to determine whether the graft induced dyskinesias observed in earlier trials can be predicted and thus avoided by appropriate selection of cells, patients and graft sites (Barker, 2019). However, human fetal material does not represent a feasible source of cells for the large-scale transplantation required if this is to become a realistic treatment for such a common neurodegenerative disease. For this reason, there has been considerable interest in the development of protocols that enable the generation of dopaminergic neurons from pluripotent stem cells, thus providing a potentially limitless number of cells for therapy.

The discovery that dopaminergic neurons arise from midbrain floor plate-derived progenitor cells has led to protocols that recapitulate the different developmental steps by promoting the specification, proliferation and differentiation of these cells (Arenas et al., 2015). These enable the generation of sufficient cells for animal-based transplantation studies and, potentially, human clinical trials using morphogens, growth factors and small molecule regulators of wnt signalling to achieve the necessary transformation from pluripotency to dopaminergic neuronal identity (Parmar et al., 2020). An important set of developmental cues that are not included, however, are the extracellular matrix molecules present in the midbrain during development. As we showed recently different laminin isoforms are involved in the genesis of dopaminergic neurons (Ahmed et al., 2019) (Zhang et al., 2017), and RNA sequencing analyses of CNS germinal zones reveals the expression of many other extracellular matrix molecules in association with neurogenesis (Fietz et al., 2012; Pollen et al., 2015; La Manno et al., 2016). However this complexity and the instructive cues it likely provides in the midbrain are not reflected in a current protocol that uses only laminin 521 to maintain pluripotent stem cells and laminin 111 for the subsequent steps of specification, proliferation and differentiation (Nolbrant et al., 2017). There may therefore be a significant opportunity to improve the yield of the dopaminergic neurons by incorporating the correct extracellular matrix cues into the protocol.

Given our limited understanding of the effects of extracellular matrix on dopaminergic neuron formation, as compared to the literature on the role of morphogens and growth factors, an unbiased approach to the identification of suitable extracellular matrix signals is required to take advantage of this opportunity. To achieve this, and also to enable the examination of extracellular matrix combinations as occur *in vivo* where multiple molecules are present in developing midbrain, we have developed a high throughput approach based on the use of a micro-contact printer to create, on a single glass slide, over 1,000 features of potential extracellular matrix combinations. Using this to examine a panel of selected extracellular matrix molecules expressed during dopaminergic neuron formation we have identified a combination of three matricellular proteins that enhance a key early step in the differentiation of dopaminergic neurons, the formation of tyrosine hydroxlase positive neuronal cells, from human pluripotent stem cells. As matricellular proteins have not previously been systematically tested for their capacity to improve the generation of dopaminergic cells from human pluripotent stem cells, our study is important in that it adds this poorly understood class of matrix molecules to the repertoire of developmental cues to be examined in these protocols. It also, by using a novel high throughput assay for extracellular matrix effects, shows the necessity of a combinatorial approach to such an examination.

## Results

### Selection of ECM Proteins for Screening

With the Matrisome project identifying in excess of 150 ECM proteins, we first began by identifying the proteins that may be expressed in the ventral mesencephalon (VM) during dopaminergic neurogenesis. Using a RNA sequencing database of mouse midbrain development (La Manno et al., 2016), we identified 74 core ECM genes that are expressed in the mouse VM between E10.5 and E14.5. From these, we selected 19 candidate genes for which there is commercially available protein and that are expressed at five reads per kilobase of transcript per million mapped reads (RPKM) or higher (Table 1). Nine of these 19 genes are enriched specifically in the VM compared to the dorsal mesencephalon and more rostral and caudal regions of the developing neural tube. A further nine genes were differentially expressed (>2 fold change) between E10.5 and E14.5, the period of peak dopaminergic neurogenesis in the mouse. Expression of the ECM proteins was also confirmed in transcriptomic data generated from human ventral mesencephalon with a number of cell types found to express the ECM proteins (La Manno et al., 2016; Kee et al., 2017). Both the Sparc and Sparc-L1 genes are expressed by endothelial cells, pericytes and radial glia in both mouse and human datasets; whereas Nell2 is expressed more broadly amongst the progenitors in the ventral mesencephalon.

**TABLE 1:** List of ECM genes examined in the combinatorial arrays, with expression levels in the RNA sequencing study of Le Manno et al. (2016) shown in reads per kilobase of transcript per million mapped reads (RPKM). As shown, three groups were selected–those most highly expressed in the VM, those whose expression in the VM changed during development, and those highly expressed throughout development. VM: ventral midbrain; RM: rostral midbrain; L: lateral plates; DM: dorsal midbrain; CM: caudal midbrain.

Differentially expressed genes in the ventral midbrain (RPKM)					
	VM	RM	L	DM	CM
Sparc	155.4	84.2	85.4	79.5	102.9
Periostin	6.4	0.7	2.1	2.2	0.5
Tsukushi	7.0	4.2	3.6	3.7	5.6
Matrilin 2	1.9	0.2	0.3	1.3	0.3
CCN3 (NOV)	1.1	0.4	0.3	0.2	0.9
IGFBP2	178.9	62.8	65.4	145.8	62.6
LGI	11.6	3.4	3.7	1.5	4.4
Laminin alpha 5	2.1	1.6	1.0	0.6	0.9
**Differentially expressed in the VM across time (RPKM)**					
	**E11.5**	**E12.5**	**E13.5**	**E14.5**	
Sparc-L1	10.4	40.3	72.3	111.9	
Thrombospondin-3	15.4	28.0	24.9	12.0	
Neurocan	48.7	80.1	85.9	89.3	
Biglycan	12.1	12.3	22.9	11.5	
Brevican	0.1	1.2	4.9	9.9	
Fibronectin	32.0	33.9	21.8	12.2	
Vitronectin	24.0	22.0	41.5	11.5	
Prolargin	0.4	6.3	7.1	8.2	
**Constitutively expressed in ventral midbrain (RPKM)**					
	**E11.5**	**E12.5**	**E13.5**	**E14.5**	
Agrin	175.5	162.3	148.2	98.5	
Nell2	70.2	81.4	77.6	82.8	
Collagen IV	33.3	40.1	55.7	27.2	

### Generation of ECM Arrays for Cell Screening

**FIGURE 1:**
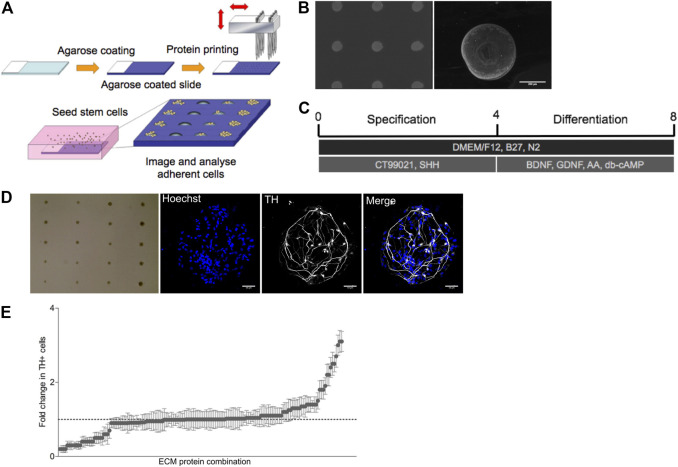
ECM array generation and differentiation of Lt-NES into TH + neurons. **(A)** Graphical outline of screening strategy with ECM arrays. **(B)** Pan-laminin antibody staining on array showing a uniform distribution of ECM proteins on approximately 200 µM spots on the agarose coated glass slide. **(C)** Differentiation protocol for human Lt-NES cells. **(D)** Brightfield image of an ECM array seeded with Lt-NES cells in culture displaying the localized growth of cells in spots seeded across the slide. Expanded fluorescent images display nuclear staining (Hoechst) and tyrosine hydroxylase (TH) a marker for dopaminergic neurons (scale bar: 50 uM). **(E)** Results from ECM array presented as fold change in the percentage of TH + neurons compared to control laminin condition (laminin derived from EHS sarcoma). The *x*-axis displays protein combinations detailed in Supplementary Table S1 ordered from those supporting least TH + neurons to those combinations with the most. Whilest the majority of combinations displayed comparable rates of differentiation as the control substrate, a small number of ECM protein combinations were able to induce greater differentiation.

The complex microenvironment created by up to 74 ECM proteins will generate multiple combinations of cell-matrix interactions that could instruct dopaminergic neurogenesis. Here we examined the role of the 19 selected ECM proteins in driving dopaminergic differentiation in an unbiased and combinatorial manner with the development of a micro-contact printing approach (Tare et al., 2009) (Figure 1A). Both single and pairwise combinations of the 19 identified ECM molecules were formed by generating an array on glass microscope slides with 190 different combinations printed with six replicates for a total of 1,140 features. Deposition of ECM proteins to the glass slide was confirmed *via* labelling with fluorescent antibodies (Figure 1B). The arrays were produced by transferring the 19 ECM proteins (at a concentration of 1 μg/ml) to a 384 well plate using a robotic liquid handler. The 384 well plates then acted as the source plates for the micro-contact printing of the ECM combinations onto an agarose coated glass slide.

### SPARC, SPARC-L1, and Nell2 Synergistically Increase the Number of TH + Neurons Derived From Neuroepithelial Progenitors

Long-term neuroepithelial stem (Lt-NES) cells (Koch et al., 2009) were seeded onto the arrays in serum free media and agitated every hour for 4 h. Prior to plating Lt-NES cells onto the combinatorial arrays, we confirmed uniformity of cell adhesion across the array using this plating protocol by using a slide printed with just one ECM protein, laminin, used in the standard culturing protocol. The cells on the combinatorial arrays were initially exposed to sonic hedgehog (Shh) and a Wnt agonist for 4 days to specify the Lt-NES cells to a ventral mesencephalic fate (Figure 1C). Thereafter, cultures were exposed to neurotrophic factors (BDNF, GDNF) as well as ascorbic acid and cyclic AMP to induce terminal differentiation. After 7 days of culture, cell were fixed and stained with antibodies against tyrosine hydroxylase (TH) to identify dopaminergic neurons (Figure 1D). The array was imaged using an Operetta high-content microscope and the number of TH + cells on each combination of ECM protein quantified using the software Columbus (Figure 1E). This analysis revealed that the majority of proteins did not increase the number of TH + neurons, while a few combinations reduced them. There were however a small number of combinations that increased the number of TH + cells; and of these, five matricellular proteins (Sparc, Sparc-L1, Nell2, trombospondin-3 (Thbs3) and neurocan (Ncan)) were represented in various combinations more than once (Supplementary Table). We concluded from this unbiased approach examining the pro-differentiation effect of ECM proteins expressed in the VM that these proteins are promising candidates for further examination.

### Matricellular Proteins Enhance Dopaminergic Neurogenesis

**FIGURE 2:**
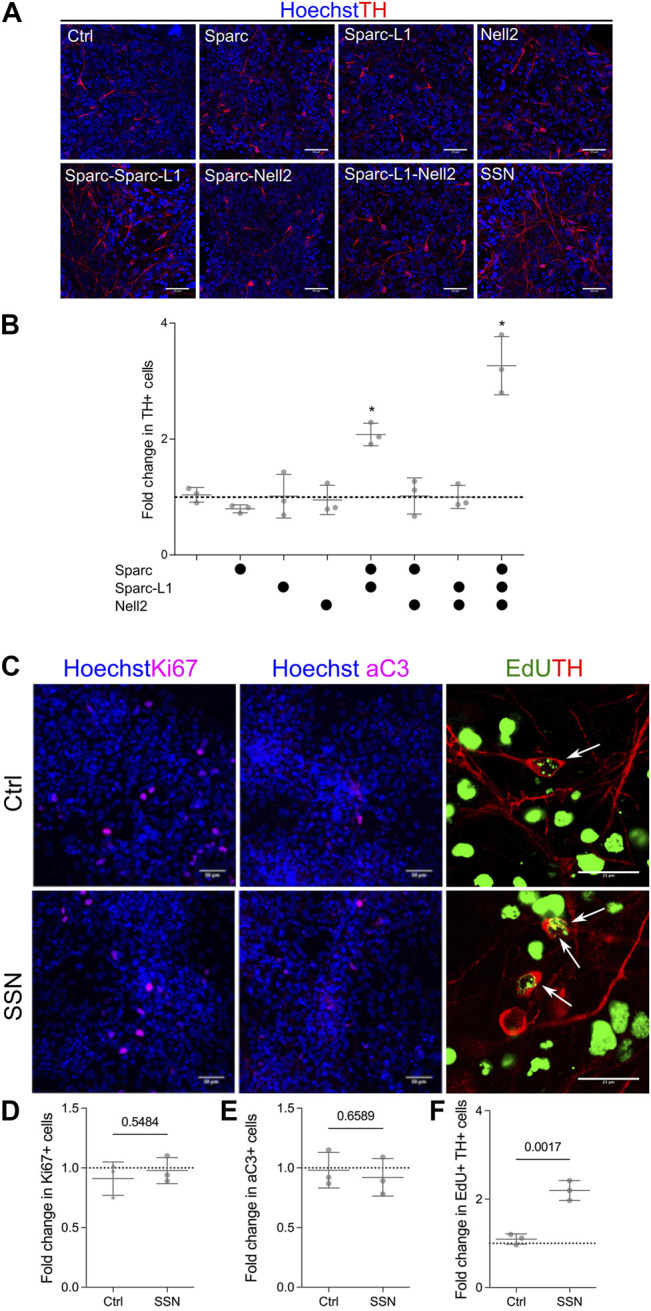
ECM arrays identify matricellular proteins that enhance the number of TH + neurons. **(A)** Immunofluorescence images of cells cultured on EHS laminin (control) or with matricellular proteins showing increased TH immunoreactivity in response to Sparc, Sparc-L1 and Nell2. **(B)** Quantification of images confirming that the synergistic action of Sparc and Sparc-L1 enhances the number of TH+ neurons and that this is further increased with the addition of Nell2 (ANOVA with Tukey’s multiple comparison test *p* < 0.0001). **(C)** Immunofluorescence images of Ki67 (proliferation), activated caspase 3 (aC3, apoptosis) and EdU/TH (neurogenesis). Matricellular proteins do not appear to impact **(D)** proliferation or **(E)** survival but do increase **(F)** neurogenesis, that is, the number of EdU and TH double positive cells (unpaired *t*-test, *p* values shown on graph). Data presented as mean fold change of percentage positive cells from three independent experiments (*N* = 3).

To validate the results seen in the array, we used standard culture platforms. Lt-NES cultures in 24 well plates were treated with the proteins either individually or in all possible combinations of the five proteins. Wells were coated with the appropriate combination of protein at a concentration of 1ug/ml per single protein. Cells were then seeded and cultured in the same manner as the ECM arrays: 4 days with media containing Shh and Wnt agonist followed by BDNF, GDNF, ascorbic acid and cyclic AMP. The validation experiments proved inconclusive for combinations involving Ncan and Thbs3. We were unable to validate the Ncan-mediated increase in differentiation, and combinations of Thbs3 combined with either Sparc or Sparc-L1 displayed inconsistent results depending on the lot of recombinant protein used. However, the experiments using Nell2, a pentameric glycoprotein that is closely related to Thbs3, and Sparc and Sparc-L1 revealed that the combination of Sparc and Sparc-L1 increased the number of TH + cells, although each protein had no effect when used on its own. Nell2 providing a further additive increase to the Sparc/Sparc-L1-driven increase in TH + neurons, resulting in a threefold increase in number as compared with cultures lacking matricellular proteins (Figures 2A,B). There was no effect of Nell2, however, when used on its own or in combination with either Sparc or Sparc-L1 suggesting a complex mechanism of action of these matricellular proteins.

Next we determined whether this increase in TH + neurons in response to Sparc, Sparc-L1 and Nell2 (a combination hereafter referred to as SSN) was a result of an increase in proliferation of the progenitors, their differentiation, or survival of the newly-generated neurons. To do this, we first examined the number of Ki67 cells (Figures 2C,D) in both control and SSN treated conditions and found no significant difference in the number of cells that remained in the cell cycle. We also examined activated caspase 3 (aC3) expression in response to SSN treatment to determine if there was a reduction in the number of apoptotic TH + neurons. Again, no significant difference was detected between control and SSN groups (Figures 2C,E). Finally, we labelled cultures with EdU on day 5 and examined the number of cells at day 8 that were positive for both EdU and TH to identify newly formed TH + neurons (ie those formed by neurogenesis from progenitors dividing at 5DIV). Here, we found a significant increase in both the number of TH + cells (7.1% vs 22,8%) and EdU + TH + double positive cells (1.3 vs 2.8%) following SSN treatment (Figures 2C,F). We conclude that SSN enhances the differentiation of VM progenitors into TH + neurons, a result that could reflect accelerated differentiation, an overall increase in neurogenesis and/or a relative increase in TH + cell differentiation.

**FIGURE 3:**
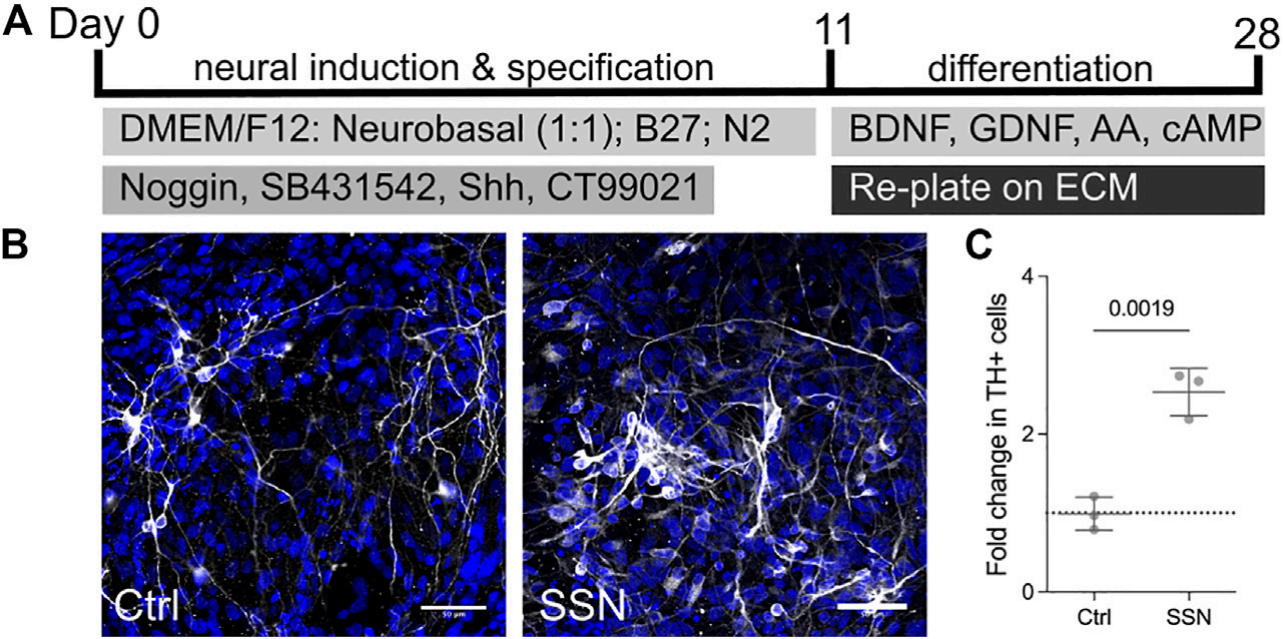
Matricellular proteins increase the number of TH + neurons derived from human embryonic stem cells. Differentiation protocol used for the dopaminergic differentiation of human embryonic stem cells. **(B)** Immunofluorescence images of neurons derived from ES cells with TH positive (white) neurons increased in the presence of matricellular proteins. **(C)** quantification of images showing a 2-fold increase in the number of TH positive neurons detected following exposure to matricellular proteins (unpaired *t*-test, *N* = 3, *p* = 0.0019).

Finally, we further examined the potential for matricellular proteins to increase the number of TH + neurons in a human embryonic stem cell-derived model that better recapitulates the human developmental trajectory and also provides direct translational relevance. Human embryonic stem cells were first induced to a neural fate and then patterned using sonic hedgehog (Shh) and a Wnt agonist as previously shown to generate rapidly dividing, FoxA2, Lmx1a and Otx2 triple positive progenitors (Nolbrant et al., 2017; Ahmed et al., 2019) (Figure 3A). Cells were replated onto wells coated with polyornithine, fibronectin and laminin as per the standard culture protocol at day 11. The matricellular proteins at an individual concentration of 1ug/ml were then added in solution as part of the culture media on day 14 and once again at day 21. The ECM molecules were added in solution so as to reduce the need for replating. Having been exposed to the matricellular proteins for a period of 14 days, cells were fixed on day 28 and examined for the number of TH positive neurons (Figure 3B). Similar to the Lt-NES cells, we found an increase (6.8 ± 0.9% on control conditions compared to 17.6 ± 2.3% following SSN treatment) in the number of TH positive cells following SSN treatment (Figure 3C).

## Discussion

The term matricellular protein was introduced by Bornstein in 1995 to describe extracellular matrix proteins such as SPARC and the thrombospondins that do not contribute to matrix structure in contrast to classical extracellular matrix molecules such as the collagens, laminins and fibronectin (Bornstein, 1995). Since then a number of such proteins have been identified, many of which are expressed at significant levels during development and in association with tumours (Bornstein and Sage, 2002). Despite this and the identification of multiple interactions, including binding to integrins and both direct and indirect effects on growth factor signalling (Murphy-Ullrich and Sage, 2014), their function remains poorly understood. Here we have identified three as promoting differentiation and the number of TH + neurons from both a human neural epithelial stem cell line and a human embryonic stem cell line; Sparc, Sparc-like, (Sparc-L1, also known as hevin), and Nell2. None have been previously linked to neural differentiation. Sparc can promote proliferation and regulate survival (Schiemann et al., 2003; Bradshaw, 2012), neither of which are affected by the Sparc/Sparc-L/Nell2 combination identified in our experiments. Within the CNS, both Sparc and Sparc-L alter synapse number, being increased by the latter and decreased by the former (Chen et al., 2020). Nell2, which by virtue of its structural homology with the thrombospondins could be considered a matricellular protein, regulates axonal guidance in spinal cord and in the visual system (Jaworski et al., 2015; Nakamoto et al., 2019). Our findings therefore add hitherto undefined functions of these proteins, the underlying signaling mechanisms of which will require further experimentation. A critical question following on from our validation assay, where none of the matricellular proteins added in isolation had an effect, will be why a combination of proteins is required to promote differentiation. Whilst the cooperative effect of Sparc and Sparc-L1 could represent an additive effect increasing the molarity of a shared functional domain this is not the case for Nell2 as the addition of this extracellular matrix to either Sparc or Sparc-L1 had no effect in the validation assay-the effect on differentiation was only seen when it was added to both of the other two matricellular proteins.

Extracellular matrix arrays using combinations of either 5 or 38 proteins printed using a DNA spotter have been used previously to study matrix effects on cell adhesion and differentiation (Flaim et al., 2005; Malta et al., 2016). The key innovation in our work is that the panel of matrix proteins we chose was selected on the basis of their expression in the relevant region of the central nervous system, enabling us to investigate combinations of specific matrix molecules present during midbrain development. In the earlier studies, the panels were chosen to be representative of matrix molecules expressed over a broad range of physiological and pathological situations. These generic arrays would therefore be predicted to have less power to detect effects on any specific lineage. Indeed, none of the three proteins we identified were included in any of these previous studies, and so the effect we discovered would not have been identified using the more broadly representative panels.

Together these studies highlight the power of the array technology to probe the complexity of the extracellular matrix. There are however three significant caveats. First, whilst larger arrays incorporating most or all of the 300 + core matrisome proteins are clearly realistic the study of combinations will, given the overall number of the extracellular matrix spots that can be printed on a single slide, require strategies for prioritisation such as the RNAseq analysis we have used here. Second, limitations on the number of possible variations that can be included on a single slide limits the ability to study different concentrations in the assay. As a result the single concentration used (1 μg/ml) might not achieve the biologically relevant concentration for some molecules, and some effective extracellular matrix molecules might be missed. Third, the changing cell-cell interactions and the effect of extracellular matrix secretion by the cells themselves as well as post-translational modifications of ECM proteins will, over the course of the experiment, alter the microenvironment provided by each spot and so degrade the precision of the assay. We limited this by selecting a dopaminergic neuron differentiation protocol that requires only 8 days, rather than the much longer human embryonic stem cell-based protocol that would be used for clinical grade cells. We recognise however that even during this 8-day period significant changes to the matrix in each spot is likely and for this reason, as is true for all high-throughput array screens, we regard the assay as a screening tool for the generation of “hits” with validation using conventional microwell assays essential to confirm or exclude observed effects.

The identification of a combination of extracellular matrix molecules expressed during midbrain development that increased the number of TH + neurons derived from human ES cells provides a potentially important enhancement to current protocols for the generation of dopaminergic neurons for therapeutic purposes, with the differentiation of TH + cells being a key step in these protocols. Given that the current protocols have been used to guide GMP manufacturing of cells for planned first-generation clinical trials of transplantation in Parkinson’s disease, our results will not have an immediate impact on the manufacture of these cells. Their translational relevance will however be important when the protocols for the second-generation trials are being designed based on the lessons learnt from the first. Here, the increase in yield we demonstrate based on the simple addition of three matrix components provides an attractive strategy for enhancing the formation of an important intermediate cell type in the protocol. Further work is now required to determine the extent to which the ECM combination exerts this effect by increasing overall neurogenesis, and whether the combination will also enhance the further differentiation steps that generate therapeutically-relevant mesencephalic dopaminergic neurons. An examination of markers expressed in batches of human embryonic stem cell-derived dopaminergic neuroprogenitors revealed that markers of caudal VM were associated with higher *in vivo* yields of dopaminergic neurons post grafting (Kirkeby et al., 2017). This caudal region of the VM generates the mesencephalic dopaminergic neurons lost in Parkinson’s disease, while the more rostral regions generate cells of the sub thalamic nucleus that are not lost by the disease. An analysis of the embryonic 15.5 mouse brain using the Allen brain Atlas shows that Sparc-L1 and Nell2 are more highly expressed in the caudal regions of the midbrain (MA, unpublished observations), consistent with a role in specifying the mesencephalic subtype. Further work on generating GMP reagents such as synthetic peptides to mimic the signals provided by these extracellular matrix molecules could therefore be of therapeutic value both to enhance yield and also to increase subtype specificity.

## Materials and Methods

### Fabrication of ECM Arrays

Aminoalkyl functionalised glass microscope slides (S5641, Sigma) were dip coated with 2% agarose. The back of the slides were cleaned with a paper tissue and left to air dry coating-side up. Agarose slides were spotted using a Genetix Qarray^mini^ microcontact printer equipped with 32 solid aQu pins. 190 unique combinations were spotted in replicates of 6, along with alexaflour-488 as a negative control and for image alignment used for analysis. Proteins were spotted at an individual concentration of 1ug/ml. The following recombinant proteins were used: agrin, biglycan, brevican, collagen IV, fibronectin, IGFBP2, laminin-511, laminin-521, matrilin-2, neurocan, Nell2, Nov, postn, prelp, Sparc, Sparc-L1, thrombospondin-3, tsukushin, vitronectin (all purchased from R&D). Slides were stored at 4°C prior to use and were used within 48 h of fabrication.

### Long-Term Neuroepithelial Stem Cell Culture

LT-NES cells were maintained in DMEM/F12 (Invitrogen, 31331–028) maintenance medium with N2 supplement (1:100), B27 supplement (1:1,000), FGF2 (10 ng/ml, R&D), and bFGF (10 ng/ml, R&D).

For screening on ECM arrays: prior to use, ECM array slides were washes with PBS and treated with UV before seeding cells. A suspension of LT-NES cells in 1 ml of media were added to the ECM array slides placed in a 4-well rectangular culture chamber. Cells were allowed to attach for 4 h at 37°C, after which slides were washed and fresh media was added. Arrays were cultured for 4 days with N2 (1:100), B27 (1:1,000), SHH (200 ng/ml, R&D), and CT99021 (1 μM, Tocris Biosciences) added to DMEM/F12 media. After that, cells were kept in media containing N2 (1:100), B27 (1:100), GDNF (20 ng/ml, R&D), BDNF (20 ng/ml, R&D), cyclic AMP (0.5mM, Sigma, D0627) and ascorbic acid (200uM, Sigma A4403) until fixation, at which point slides were washed with PBS and fixed with 3.7% PFA for 15 min.

For validation studies, LT-NES cells were dissociated with TrypLE Select and seeded (100,000 cells per well) in 48‐well plates coated with polyornithine and matricellular proteins (Sparc (941-SP, R&D), Sparc-L1 (2728-SL, R&D) and Nell2 (8946-NL, R&D)) or laminin (), and cultured for 4 days with N2 (1:100), B27 (1:1,000), SHH (200 ng/ml, R&D), and CT99021 (1 μM, Tocris Biosciences). After that, cells were kept on media containing N2 (1:100), B27 (1:100), GDNF (20 ng/ml, R&D), BDNF (20 ng/ml, R&D), cyclic AMP (0.5mM, Sigma, D0627) and ascorbic acid (200uM, Sigma A4403) until fixation where they were treated with 3.7% PFA for 15 min. For EdU labelling, EdU was administered at day 5 for 24 h to capture dividing cells. Cells were then fixed at day 8 for analysis.

### Dopaminergic Differentiation of Human Embryonic Stem Cells

Undifferentiated RC17 ESCs (passages 30–58, Roslin Cells, hPSC reg. no. RCe021-A) were maintained in E8 medium (A1517001) on Geltrex (1%, 12760021)-coated plates and passaged weekly with EDTA (0.5 mM). To start differentiation (day 0), human ESC colonies were detached using EDTA (0.5 mM) and placed in non-treated 60-mm culture dishes in differentiation medium consisting of DMEM:F12/Neurobasal (1:1), N2 supplement (1:100), B27 supplement (1:50), SB431542 (10 μM, Tocris Biosciences), rhnoggin (200 ng/ml, R&D), SHH-C24II (200 ng/ml, R&D Systems), and CHIR99021 (0.8 μM, Tocris Biosciences). Medium was changed once on day 2. The resultant embryoid bodies were collected on day 4 and placed on polyornithine (PO)-, fibronectin (Fn)-, and laminin (Lm)-coated plates in reduced N2 (1:200) and B27 (1:100) condition. Growth and patterning factors were removed on day 9 with the cultures kept in DMEM:F12/Neurobasal (1:1), N2 supplement (1:200), and B27 supplement (1:100). On day 11 of differentiation, the cell clusters were dissociated to single cells with accutase and replated onto dry PO/Fn/Lm-coated plates in Neurobasal, B27 (1:50), BDNF (20 ng/ml), GDNF (10 ng/ml), AA (200 μM), and db-cAMP (0.5 mM, Sigma). From day 14, cultures were treated with matricellular proteins Sparc, Sparc-L1 and Nell-2 (1 μg/ml, R&D Systems) up until day 28, when they were fixed in 3.7% PFA for 15 min.

### Immunocytochemistry, Microscopy and Image Analysis

For both the cells on the arrays and the validation experiments in standard culture plates, cells were washed with PBS and blocked in PBTA (5% normal goat/donkey serum, 0.1% Triton X-100, 1% BSA) for 1 h at room temperature. Primary antibodies were incubated in PBTA and staining was carried out overnight at 4°C. Antibodies used were cleaved caspase 3 (1:200, Cell Signaling Technology, 9661S); TH (1:1,000, Millipore, AB152); Ki67 (1:500, abcam, ab15580); Click-iT EdU (Invitrogen, C10337). Corresponding secondary antibodies were Alexa Fluor Dyes (Invitrogen) and used at a dilution of 1:1,000 for 1 h at room temperature. Cells were counterstained with Hoechst 33258 (Sigma). For validation studies where cells are in culture plates, these were washed and kept in PBS. The arrays were washed and mounted with Fluoromount G Mounting Media (Thermo). Images were captured on the Operetta high-content imaging system (Perkin Elmer) or a Lecia SP8 confocal microscope (Leica). For the ECM arrays, the whole slide was scanned and images stitched together using the Columbus software (Perkin Elmer). Image quantification was conducted using an automated image analysis software package (Columbus, Perkin Elmer) using a pre-validated pipeline that quantified nuclei and cytoplasmic TH expression. Image analysis for validation studies was performed on five images per well, three wells per condition across three experimental repeats (*n* = 3). Cell counts were performed and percent positive cells for each marker was determined and then normalized to the control condition, with data presented as mean fold change ±standard deviation.

## Data Availability

The original contributions presented in the study are included in the article/Supplementary Material, further inquiries can be directed to the corresponding author.
