# Supplementary material for: Combinatorial ECM Arrays Identify Cooperative Roles for Matricellular Proteins in Enhancing the Generation of TH+ Neurons From Human Pluripotent Cells
Source: Front Cell Dev Biol. 2021 Dec 1;9:755406. doi: 10.3389/fcell.2021.755406 (PMC8672163; doi:10.3389/fcell.2021.755406)
Supplement: Supplementary file 1 [file Table1.DOCX]

| **Protein combination** | | | **No of cells** | | | |  | **TH+ cells** | | |  | **% diffn (%)** | | |
| --- | --- | --- | --- | --- | --- | --- | --- | --- | --- | --- | --- | --- | --- | --- |
| Laminin control (EHS carcinoma) | | | 203 | | ± | 85.4 |  | 24 | ± | 3.3 |  | 11.8 | ± | 3.9 |
|  |  | |  | |  |  |  |  |  |  |  |  |  |  |
| Sparc-L1 | | Sparc | | 161 | ± | 54.7 |  | 52 | ± | 6.5 |  | 32.3 | ± | 11.9 |
| Sparc | | Nell2 | | 41 | ± | 8.2 |  | 13 | ± | 1 |  | 31.7 | ± | 12.2 |
| Thrombospondin-3 | | Sparc | | 72 | ± | 8.6 |  | 22 | ± | 1 |  | 30.6 | ± | 11.6 |
| Thrombospondin-3 | | Sparc-L1 | | 51 | ± | 7.1 |  | 14 | ± | 0.7 |  | 27.5 | ± | 9.9 |
| Sparc-L1 | | Nell2 | | 59 | ± | 24.1 |  | 16 | ± | 2.4 |  | 27.1 | ± | 10.0 |
| Sparc | | Neurocan | | 67 | ± | 30 |  | 17 | ± | 2.8 |  | 25.4 | ± | 9.3 |
| Thrombospondin-3 | | Nell2 | | 53 | ± | 9.5 |  | 13 | ± | 0.9 |  | 24.5 | ± | 9.5 |
| Sparc-L1 | | Neurocan | | 74 | ± | 8.9 |  | 17 | ± | 0.8 |  | 23.0 | ± | 9.0 |
| Vitronectin | | Neurocan | | 53 | ± | 7.5 |  | 10 | ± | 0.5 |  | 18.9 | ± | 6.7 |
| Vitronectin | | Nell2 | | 17 | ± | 3 |  | 3 | ± | 0.2 |  | 17.6 | ± | 6.7 |
| Agrin | | Prelp | | 37 | ± | 11.9 |  | 6 | ± | 0.7 |  | 16.2 | ± | 5.9 |
| Nell2 | | Neurocan | | 19 | ± | 3.4 |  | 3 | ± | 0.2 |  | 15.8 | ± | 5.9 |
| Agrin | | Nell2 | | 38 | ± | 10.2 |  | 6 | ± | 0.6 |  | 15.8 | ± | 5.9 |
| Prelp | | Matrilin-2 | | 44 | ± | 5.8 |  | 7 | ± | 0.3 |  | 15.9 | ± | 5.2 |
| Fibronectin | | Thrombospondin-3 | | 133 | ± | 39.5 |  | 21 | ± | 2.3 |  | 15.8 | ± | 5.8 |
| Tsukushin | | Nell2 | | 13 | ± | 1.8 |  | 2 | ± | 0.1 |  | 15.4 | ± | 5.6 |
| Postn | | Neurocan | | 34 | ± | 13.7 |  | 5 | ± | 0.8 |  | 14.7 | ± | 5.8 |
|  | |  | |  |  |  |  |  |  |  |  |  |  |  |
| Sparc | |  | | 108 | ± | 45.6 |  | 14 | ± | 2.5 |  | 13.0 | ± | 5.5 |
| Sparc | | Nov | | 29 | ± | 15.7 |  | 4 | ± | 0.8 |  | 13.8 | ± | 5.1 |
| Brevican | | Biglycan | | 160 | ± | 35.2 |  | 22 | ± | 1.8 |  | 13.8 | ± | 5.1 |
| IGFBP2 | | Agrin | | 30 | ± | 8.5 |  | 4 | ± | 0.4 |  | 13.3 | ± | 4.7 |
| Vitronectin | |  | | 132 | ± | 22.4 |  | 17 | ± | 1.1 |  | 12.9 | ± | 4.9 |
| Tsukushin | | Sparc-L1 | | 24 | ± | 4.1 |  | 3 | ± | 0.2 |  | 12.5 | ± | 4.9 |
| IGFBP2 | | Biglycan | | 48 | ± | 23.1 |  | 6 | ± | 1.1 |  | 12.5 | ± | 4.8 |
| Matrilin-2 | | Lm-521 | | 106 | ± | 48.3 |  | 13 | ± | 2.2 |  | 12.3 | ± | 4.6 |
| Prelp | |  | | 122 | ± | 25.7 |  | 15 | ± | 1.2 |  | 12.3 | ± | 4.7 |
| Thrombospondin-3 | | Lm-521 | | 16 | ± | 2.5 |  | 2 | ± | 0.1 |  | 12.5 | ± | 4.0 |
| Matrilin-2 | |  | | 132 | ± | 35.5 |  | 16 | ± | 1.6 |  | 12.1 | ± | 4.5 |
| Sparc-L1 | |  | | 108 | ± | 15.1 |  | 13 | ± | 0.7 |  | 12.0 | ± | 4.6 |
| Collagen IV | | Brevican | | 160 | ± | 36.8 |  | 19 | ± | 1.6 |  | 11.9 | ± | 4.3 |
| Matrilin-2 | | Fibronectin | | 118 | ± | 21.3 |  | 14 | ± | 0.9 |  | 11.9 | ± | 4.2 |
| Agrin | | Vitronectin | | 127 | ± | 15.2 |  | 15 | ± | 0.7 |  | 11.8 | ± | 4.6 |
| Sparc | | Prelp | | 60 | ± | 9.4 |  | 7 | ± | 0.4 |  | 11.7 | ± | 4.3 |
| Fibronectin | | Brevican | | 60 | ± | 7.8 |  | 7 | ± | 0.3 |  | 11.7 | ± | 3.8 |
| Biglycan | | Postn | | 43 | ± | 7.3 |  | 5 | ± | 0.3 |  | 11.6 | ± | 4.1 |
| Agrin | | Postn | | 69 | ± | 34.8 |  | 8 | ± | 1.5 |  | 11.6 | ± | 4.3 |
| Agrin | |  | | 113 | ± | 45.9 |  | 13 | ± | 2 |  | 11.5 | ± | 4.4 |
| Fibronectin | |  | | 226 | ± | 81.2 |  | 26 | ± | 3.5 |  | 11.5 | ± | 4.3 |
| Neurocan | | Biglycan | | 78 | ± | 11 |  | 9 | ± | 0.5 |  | 11.5 | ± | 4.5 |
| Lm-511 | | Thrombospondin-3 | | 78 | ± | 36 |  | 9 | ± | 1.5 |  | 11.5 | ± | 4.2 |
| Fibronectin | | Tsukushin | | 141 | ± | 25.3 |  | 16 | ± | 1.1 |  | 11.3 | ± | 4.3 |
| Prelp | | Neurocan | | 53 | ± | 7.9 |  | 6 | ± | 0.3 |  | 11.3 | ± | 3.8 |
| Thrombospondin-3 | | Nov | | 27 | ± | 9.7 |  | 3 | ± | 0.4 |  | 11.1 | ± | 4.1 |
| Biglycan | | Prelp | | 80 | ± | 12.5 |  | 9 | ± | 0.5 |  | 11.3 | ± | 4.0 |
| Nov | |  | | 80 | ± | 28 |  | 9 | ± | 1.2 |  | 11.3 | ± | 4.3 |
| Postn | | Matrilin-2 | | 62 | ± | 22.5 |  | 7 | ± | 0.9 |  | 11.3 | ± | 4.0 |
| Brevican | | Tsukushin | | 72 | ± | 51.1 |  | 8 | ± | 2.1 |  | 11.1 | ± | 4.1 |
| Vitronectin | | Nov | | 90 | ± | 49.1 |  | 10 | ± | 2 |  | 11.1 | ± | 4.1 |
| IGFBP2 | | Thrombospondin-3 | | 73 | ± | 16.8 |  | 8 | ± | 0.7 |  | 11.0 | ± | 4.2 |
| Lm-511 | | Fibronectin | | 37 | ± | 6.6 |  | 4 | ± | 0.3 |  | 10.8 | ± | 4.5 |
| Lm-521 | | IGFBP2 | | 46 | ± | 6.4 |  | 5 | ± | 0.3 |  | 10.9 | ± | 4.7 |
| Matrilin-2 | | Lm-511 | | 55 | ± | 7.2 |  | 6 | ± | 0.3 |  | 10.9 | ± | 4.2 |
| Nell2 | | Matrilin-2 | | 46 | ± | 5.5 |  | 5 | ± | 0.2 |  | 10.9 | ± | 3.6 |
| Postn | | Nell2 | | 46 | ± | 6 |  | 5 | ± | 0.2 |  | 10.9 | ± | 3.3 |
| Matrilin-2 | | IGFBP2 | | 83 | ± | 0 |  | 9 | ± | 0 |  | 10.8 | ± | 0.0 |
| Agrin | | Nov | | 65 | ± | 15.5 |  | 7 | ± | 0.6 |  | 10.8 | ± | 3.9 |
| Collagen IV | | Sparc | | 111 | ± | 29.9 |  | 12 | ± | 1.2 |  | 10.8 | ± | 4.0 |
| Lm-511 | | Agrin | | 111 | ± | 13.3 |  | 12 | ± | 0.5 |  | 10.8 | ± | 3.8 |
| IGFBP2 | | Tsukushin | | 74 | ± | 37.7 |  | 8 | ± | 1.5 |  | 10.8 | ± | 4.0 |
| Fibronectin | | Sparc-L1 | | 139 | ± | 30.7 |  | 15 | ± | 1.2 |  | 10.8 | ± | 3.9 |
| Sparc | | IGFBP2 | | 139 | ± | 22.3 |  | 15 | ± | 0.9 |  | 10.8 | ± | 4.0 |
| Lm-521 | | Collagen IV | | 149 | ± | 20.8 |  | 16 | ± | 0.8 |  | 10.7 | ± | 3.8 |
| Prelp | | Lm-511 | | 112 | ± | 39.1 |  | 12 | ± | 1.6 |  | 10.7 | ± | 4.1 |
| Lm-521 | | Tsukushin | | 84 | ± | 27.8 |  | 9 | ± | 1.1 |  | 10.7 | ± | 4.0 |
| IGFBP2 | | Vitronectin | | 178 | ± | 32 |  | 19 | ± | 1.3 |  | 10.7 | ± | 4.1 |
| Nell2 | | Biglycan | | 47 | ± | 6.6 |  | 5 | ± | 0.3 |  | 10.6 | ± | 4.5 |
| Neurocan | | Collagen IV | | 225 | ± | 51.8 |  | 24 | ± | 2 |  | 10.7 | ± | 3.9 |
| Tsukushin | |  | | 66 | ± | 15.8 |  | 7 | ± | 0.6 |  | 10.6 | ± | 3.8 |
| Prelp | | Postn | | 47 | ± | 23.8 |  | 5 | ± | 0.9 |  | 10.6 | ± | 3.8 |
| Biglycan | | Agrin | | 85 | ± | 22.8 |  | 9 | ± | 0.9 |  | 10.6 | ± | 3.9 |
| Nov | | Nell2 | | 28 | ± | 13.4 |  | 3 | ± | 0.5 |  | 10.7 | ± | 3.7 |
| Fibronectin | | Collagen IV | | 198 | ± | 31.6 |  | 21 | ± | 1.2 |  | 10.6 | ± | 3.8 |
| Matrilin-2 | | Biglycan | | 85 | ± | 10.8 |  | 9 | ± | 0.4 |  | 10.6 | ± | 3.7 |
| Thrombospondin-3 | | Neurocan | | 85 | ± | 35.8 |  | 9 | ± | 1.4 |  | 10.6 | ± | 3.9 |
| Sparc-L1 | | Lm-521 | | 113 | ± | 43.2 |  | 12 | ± | 1.7 |  | 10.6 | ± | 3.9 |
| Nov | | Brevican | | 86 | ± | 11.2 |  | 9 | ± | 0.4 |  | 10.5 | ± | 3.6 |
| Collagen IV | | Thrombospondin-3 | | 181 | ± | 69.5 |  | 19 | ± | 2.7 |  | 10.5 | ± | 3.9 |
| Lm-511 | | IGFBP2 | | 124 | ± | 14.9 |  | 13 | ± | 0.6 |  | 10.5 | ± | 4.0 |
| Biglycan | | Tsukushin | | 57 | ± | 15.7 |  | 6 | ± | 0.6 |  | 10.5 | ± | 3.8 |
| Nov | | Collagen IV | | 115 | ± | 17.2 |  | 12 | ± | 0.7 |  | 10.4 | ± | 4.1 |
| Sparc-L1 | | Lm-511 | | 57 | ± | 8 |  | 6 | ± | 0.3 |  | 10.5 | ± | 3.8 |
| Brevican | | Vitronectin | | 86 | ± | 24.7 |  | 9 | ± | 1 |  | 10.5 | ± | 4.0 |
| Nell2 | | Lm-521 | | 77 | ± | 10 |  | 8 | ± | 0.4 |  | 10.4 | ± | 4.0 |
| Thrombospondin-3 | | Prelp | | 115 | ± | 36.9 |  | 12 | ± | 1.4 |  | 10.4 | ± | 3.8 |
| Fibronectin | | Agrin | | 115 | ± | 47.1 |  | 12 | ± | 1.8 |  | 10.4 | ± | 3.8 |
| Lm-511 | | Brevican | | 115 | ± | 16.1 |  | 12 | ± | 0.6 |  | 10.4 | ± | 3.7 |
| Nov | | Lm-521 | | 77 | ± | 10.8 |  | 8 | ± | 0.4 |  | 10.4 | ± | 3.7 |
| Sparc-L1 | | Nov | | 77 | ± | 10 |  | 8 | ± | 0.4 |  | 10.4 | ± | 4.0 |
| Tsukushin | | Prelp | | 48 | ± | 18.3 |  | 5 | ± | 0.7 |  | 10.4 | ± | 3.8 |
| Tsukushin | | Sparc | | 67 | ± | 11.4 |  | 7 | ± | 0.4 |  | 10.4 | ± | 3.5 |
| Biglycan | | Nov | | 29 | ± | 2.9 |  | 3 | ± | 0.1 |  | 10.3 | ± | 3.4 |
| Vitronectin | | Thrombospondin-3 | | 107 | ± | 28.8 |  | 11 | ± | 1.1 |  | 10.3 | ± | 3.8 |
| IGFBP2 | | Fibronectin | | 175 | ± | 47.2 |  | 18 | ± | 1.8 |  | 10.3 | ± | 3.8 |
| Lm-511 | | Biglycan | | 175 | ± | 8 |  | 18 | ± | 0.3 |  | 10.3 | ± | 3.8 |
| Matrilin-2 | | Brevican | | 78 | ± | 17.1 |  | 8 | ± | 0.7 |  | 10.3 | ± | 4.1 |
| Biglycan | | Sparc | | 58 | ± | 9.9 |  | 6 | ± | 0.4 |  | 10.3 | ± | 4.0 |
| Nov | | IGFBP2 | | 58 | ± | 15.7 |  | 6 | ± | 0.6 |  | 10.3 | ± | 3.8 |
| Sparc | | Matrilin-2 | | 58 | ± | 26.6 |  | 6 | ± | 1 |  | 10.3 | ± | 3.8 |
| Matrilin-2 | | Vitronectin | | 137 | ± | 20.5 |  | 14 | ± | 0.8 |  | 10.2 | ± | 3.9 |
| Lm-521 | | Agrin | | 108 | ± | 36.6 |  | 11 | ± | 1.4 |  | 10.2 | ± | 3.8 |
| Prelp | | Lm-521 | | 127 | ± | 24.2 |  | 13 | ± | 0.9 |  | 10.2 | ± | 3.7 |
| Biglycan | |  | | 118 | ± | 40 |  | 12 | ± | 1.5 |  | 10.2 | ± | 3.8 |
| Thrombospondin-3 | |  | | 118 | ± | 63.9 |  | 12 | ± | 2.4 |  | 10.2 | ± | 3.8 |
| Sparc | | Lm-521 | | 69 | ± | 32.7 |  | 7 | ± | 1.2 |  | 10.1 | ± | 3.7 |
| Neurocan | | Lm-521 | | 69 | ± | 11.7 |  | 7 | ± | 0.4 |  | 10.1 | ± | 3.4 |
| Nov | | Neurocan | | 69 | ± | 6.9 |  | 7 | ± | 0.3 |  | 10.1 | ± | 4.3 |
| Lm-521 | | Fibronectin | | 138 | ± | 20.7 |  | 14 | ± | 0.8 |  | 10.1 | ± | 3.9 |
| Collagen IV | | Agrin | | 70 | ± | 3.2 |  | 7 | ± | 0.1 |  | 10.0 | ± | 3.1 |
| Brevican | | Agrin | | 110 | ± | 23.2 |  | 11 | ± | 0.9 |  | 10.0 | ± | 3.9 |
| Vitronectin | | Tsukushin | | 101 | ± | 18.2 |  | 10 | ± | 0.7 |  | 9.9 | ± | 3.8 |
| Postn | | Nov | | 61 | ± | 22.1 |  | 6 | ± | 0.8 |  | 9.8 | ± | 3.6 |
| Neurocan | | IGFBP2 | | 82 | ± | 48 |  | 8 | ± | 1.7 |  | 9.8 | ± | 3.5 |
| Sparc-L1 | | Prelp | | 92 | ± | 33.5 |  | 9 | ± | 1.2 |  | 9.8 | ± | 3.6 |
| Postn | | Fibronectin | | 103 | ± | 10.3 |  | 10 | ± | 0.4 |  | 9.7 | ± | 3.9 |
| Lm-521 | | Lm-511 | | 133 | ± | 25.3 |  | 13 | ± | 0.9 |  | 9.8 | ± | 3.6 |
| Thrombospondin-3 | | Postn | | 72 | ± | 35.3 |  | 7 | ± | 1.3 |  | 9.7 | ± | 3.7 |
| Brevican | | Sparc | | 52 | ± | 17.5 |  | 5 | ± | 0.6 |  | 9.6 | ± | 3.4 |
| Collagen IV | | Sparc-L1 | | 103 | ± | 14.4 |  | 10 | ± | 0.5 |  | 9.7 | ± | 3.5 |
| Agrin | | Tsukushin | | 41 | ± | 10.8 |  | 4 | ± | 0.4 |  | 9.8 | ± | 3.7 |
| Sparc | | Postn | | 83 | ± | 31.6 |  | 8 | ± | 1.1 |  | 9.6 | ± | 3.5 |
| Lm-511 | | Vitronectin | | 125 | ± | 44.9 |  | 12 | ± | 1.6 |  | 9.6 | ± | 3.6 |
| Biglycan | | Thrombospondin-3 | | 62 | ± | 11.2 |  | 6 | ± | 0.4 |  | 9.7 | ± | 3.6 |
| IGFBP2 | | Brevican | | 94 | ± | 43.4 |  | 9 | ± | 1.5 |  | 9.6 | ± | 3.5 |
| Lm-521 | | Biglycan | | 126 | ± | 51.5 |  | 12 | ± | 1.8 |  | 9.5 | ± | 3.5 |
| Neurocan | |  | | 136 | ± | 56.2 |  | 13 | ± | 2 |  | 9.6 | ± | 3.6 |
| IGFBP2 | | Collagen IV | | 147 | ± | 47.7 |  | 14 | ± | 1.7 |  | 9.5 | ± | 3.6 |
| Prelp | | Nov | | 74 | ± | 36.1 |  | 7 | ± | 1.3 |  | 9.5 | ± | 3.6 |
| Matrilin-2 | | Collagen IV | | 221 | ± | 101.6 |  | 21 | ± | 3.6 |  | 9.5 | ± | 3.5 |
| Lm-511 | | Collagen IV | | 74 | ± | 24.4 |  | 7 | ± | 0.9 |  | 9.5 | ± | 3.7 |
| Prelp | | Nell2 | | 53 | ± | 19.3 |  | 5 | ± | 0.7 |  | 9.4 | ± | 3.6 |
| Sparc-L1 | | Postn | | 53 | ± | 22.1 |  | 5 | ± | 0.8 |  | 9.4 | ± | 3.6 |
| Vitronectin | | Postn | | 127 | ± | 45.9 |  | 12 | ± | 1.6 |  | 9.4 | ± | 3.5 |
| Tsukushin | | Postn | | 74 | ± | 40.5 |  | 7 | ± | 1.4 |  | 9.5 | ± | 3.5 |
| Postn | | Lm-521 | | 244 | ± | 34.2 |  | 23 | ± | 1.2 |  | 9.4 | ± | 3.5 |
| Postn | | Collagen IV | | 160 | ± | 72 |  | 15 | ± | 2.5 |  | 9.4 | ± | 3.5 |
| Postn | |  | | 118 | ± | 55.8 |  | 11 | ± | 1.9 |  | 9.3 | ± | 3.4 |
| Vitronectin | | Sparc-L1 | | 139 | ± | 75.9 |  | 13 | ± | 2.6 |  | 9.4 | ± | 3.4 |
| Neurocan | | Agrin | | 43 | ± | 6 |  | 4 | ± | 0.2 |  | 9.3 | ± | 3.3 |
| Collagen IV | | Biglycan | | 64 | ± | 11.6 |  | 6 | ± | 0.4 |  | 9.4 | ± | 3.4 |
| Lm-521 | | Brevican | | 97 | ± | 11.2 |  | 9 | ± | 0.4 |  | 9.3 | ± | 3.6 |
| Collagen IV | | Vitronectin | | 173 | ± | 20.7 |  | 16 | ± | 0.7 |  | 9.2 | ± | 3.4 |
| Fibronectin | | Sparc | | 140 | ± | 6.4 |  | 13 | ± | 0.2 |  | 9.3 | ± | 3.1 |
| Nov | | Lm-511 | | 141 | ± | 26.7 |  | 13 | ± | 0.9 |  | 9.2 | ± | 3.4 |
| Collagen IV | | Prelp | | 98 | ± | 27.9 |  | 9 | ± | 1 |  | 9.2 | ± | 3.6 |
| Neurocan | | Lm-511 | | 163 | ± | 22.8 |  | 15 | ± | 0.8 |  | 9.2 | ± | 3.5 |
| Sparc | | Lm-511 | | 109 | ± | 53.5 |  | 10 | ± | 1.8 |  | 9.2 | ± | 3.4 |
| Agrin | | Thrombospondin-3 | | 66 | ± | 6.6 |  | 6 | ± | 0.2 |  | 9.1 | ± | 3.0 |
| Nell2 | | Collagen IV | | 120 | ± | 32.5 |  | 11 | ± | 1.1 |  | 9.2 | ± | 3.4 |
| Nell2 | | Brevican | | 99 | ± | 35.5 |  | 9 | ± | 1.2 |  | 9.1 | ± | 3.4 |
| Neurocan | | Matrilin-2 | | 66 | ± | 7.9 |  | 6 | ± | 0.3 |  | 9.1 | ± | 3.8 |
| Lm-511 | | Tsukushin | | 99 | ± | 15.8 |  | 9 | ± | 0.5 |  | 9.1 | ± | 3.2 |
| Tsukushin | | Thrombospondin-3 | | 55 | ± | 28.6 |  | 5 | ± | 1 |  | 9.1 | ± | 3.5 |
| Biglycan | | Vitronectin | | 110 | ± | 21 |  | 10 | ± | 0.7 |  | 9.1 | ± | 3.3 |
| Nell2 | | Lm-511 | | 67 | ± | 30.9 |  | 6 | ± | 1 |  | 9.0 | ± | 3.2 |
| Postn | | Lm-511 | | 191 | ± | 22.9 |  | 17 | ± | 0.8 |  | 8.9 | ± | 3.5 |
| Sparc-L1 | | Matrilin-2 | | 90 | ± | 18.9 |  | 8 | ± | 0.6 |  | 8.9 | ± | 3.2 |
| Brevican | |  | | 169 | ± | 50.2 |  | 15 | ± | 1.6 |  | 8.9 | ± | 3.2 |
| Tsukushin | | Neurocan | | 12 | ± | 1.4 |  | 1 | ± | 0 |  | 8.3 | ± | 0.0 |
| Fibronectin | | Biglycan | | 94 | ± | 10.9 |  | 8 | ± | 0.3 |  | 8.5 | ± | 2.8 |
| Nell2 | |  | | 99 | ± | 23.7 |  | 8 | ± | 0.7 |  | 8.1 | ± | 3.0 |
| IGFBP2 | |  | | 75 | ± | 25.6 |  | 6 | ± | 0.8 |  | 8.0 | ± | 3.1 |
| Lm-511 | |  | | 193 | ± | 24.6 |  | 15 | ± | 0.7 |  | 7.8 | ± | 2.8 |
| Vitronectin | | Sparc | | 14 | ± | 5.2 |  | 1 | ± | 0.1 |  | 7.1 | ± | 1.9 |
| Tsukushin | | Nov | | 90 | ± | 42.9 |  | 6 | ± | 1.1 |  | 6.7 | ± | 2.6 |
| Collagen IV | | Tsukushin | | 159 | ± | 15.9 |  | 10 | ± | 0.4 |  | 6.3 | ± | 2.5 |
| Prelp | | Fibronectin | | 168 | ± | 25.2 |  | 10 | ± | 0.6 |  | 6.0 | ± | 2.4 |
| Lm-521 | |  | | 183 | ± | 74.9 |  | 10 | ± | 1.5 |  | 5.5 | ± | 2.0 |
| Collagen IV | |  | | 202 | ± | 102.7 |  | 11 | ± | 2.1 |  | 5.4 | ± | 2.0 |
| Matrilin-2 | | Agrin | | 77 | ± | 35.6 |  | 4 | ± | 0.7 |  | 5.2 | ± | 2.0 |
| Postn | | IGFBP2 | | 58 | ± | 14 |  | 3 | ± | 0.3 |  | 5.2 | ± | 2.1 |
| Vitronectin | | Prelp | | 80 | ± | 33.3 |  | 4 | ± | 0.6 |  | 5.0 | ± | 1.8 |
| Brevican | | Prelp | | 60 | ± | 7.2 |  | 3 | ± | 0.1 |  | 5.0 | ± | 1.4 |
| Fibronectin | | Vitronectin | | 304 | ± | 83 |  | 15 | ± | 1.5 |  | 4.9 | ± | 1.8 |
| Biglycan | | Sparc-L1 | | 44 | ± | 7.5 |  | 2 | ± | 0.1 |  | 4.5 | ± | 1.3 |
| Brevican | | Postn | | 98 | ± | 26.7 |  | 4 | ± | 0.4 |  | 4.1 | ± | 1.5 |
| Tsukushin | | Matrilin-2 | | 25 | ± | 4.4 |  | 1 | ± | 0.1 |  | 4.0 | ± | 2.3 |
| Nell2 | | Fibronectin | | 154 | ± | 24.6 |  | 6 | ± | 0.4 |  | 3.9 | ± | 1.6 |
| Agrin | | Sparc-L1 | | 53 | ± | 20.2 |  | 2 | ± | 0.3 |  | 3.8 | ± | 1.5 |
| Nov | | Fibronectin | | 108 | ± | 44.3 |  | 4 | ± | 0.6 |  | 3.7 | ± | 1.4 |
| Neurocan | | Fibronectin | | 55 | ± | 18.2 |  | 2 | ± | 0.2 |  | 3.6 | ± | 1.1 |
| Brevican | | Thrombospondin-3 | | 56 | ± | 15 |  | 2 | ± | 0.2 |  | 3.6 | ± | 1.3 |
| Brevican | | Sparc-L1 | | 32 | ± | 17.3 |  | 1 | ± | 0.2 |  | 3.1 | ± | 1.2 |
| Lm-521 | | Vitronectin | | 99 | ± | 26.7 |  | 3 | ± | 0.3 |  | 3.0 | ± | 1.1 |
| IGFBP2 | | Sparc-L1 | | 67 | ± | 7.7 |  | 2 | ± | 0.1 |  | 3.0 | ± | 1.3 |
| Nov | | Matrilin-2 | | 67 | ± | 30.2 |  | 2 | ± | 0.3 |  | 3.0 | ± | 1.0 |
| Neurocan | | Brevican | | 69 | ± | 28.2 |  | 2 | ± | 0.3 |  | 2.9 | ± | 1.1 |
| Nell2 | | IGFBP2 | | 37 | ± | 5.6 |  | 1 | ± | 0.1 |  | 2.7 | ± | 1.8 |
| Thrombospondin-3 | | Matrilin-2 | | 41 | ± | 14.8 |  | 1 | ± | 0.1 |  | 2.4 | ± | 0.7 |
| Prelp | | IGFBP2 | | 99 | ± | 12.8 |  | 2 | ± | 0.1 |  | 2.0 | ± | 0.8 |
| Agrin | | Sparc | | 58 | ± | 6.9 |  | 1 | ± | 0 |  | 1.7 | ± | 0.0 |

**Table S1**: A list of the ECM protein combinations examined and the number of total cells and TH+ neurons identified per condition. Data is presented as the average of replicates ± standard deviation, and is ranked by the % TH+ cells in the total number (No) of cells. The differentiation induced by the EHS laminin control is show first, then the 17 combinations that generated greater levels of differentiation than EHS laminin. Note, as highlighted by the colouring, that 5 matricellular proteins generate the best 8 combinations and are found 4 or more times within the 17 combinations, as a result of which they were selected for validation studies. Combinations below the blank line in the Table generated a % TH+ cell count below that of the laminin control, although in some of these the total number of cells was higher.
